# Shortening telomere is associated with subclinical atherosclerosis biomarker in omnivorous but not in vegetarian healthy men

**DOI:** 10.18632/aging.102098

**Published:** 2019-07-19

**Authors:** Naiara Cinegaglia, Luiza Antoniazzi, Daniela Rosa, Debora Miranda, Julio Acosta-Navarro, Luiz Bortolotto, Valeria Hong, Valeria Sandrim

**Affiliations:** 1 Institute of Bioscience, São Paulo State University – IBB/UNESP, Botucatu, São Paulo, Brazil; 2 Heart Institute (InCor), Medical School, University of São Paulo, São Paulo, Brazil; 3 Laboratory of Molecular Medicine, Medical School, Federal University of Minas Gerais, UFMG, Minas Gerais, Brazil

**Keywords:** vegetarian, telomere length, carotid intima-media thickness, cardiovascular

## Abstract

Telomere length is considered to be a biomarker of biological aging and age-related disease. There are few studies that have evaluated the association between telomere length and diet, and none of them have evaluated the impact of a vegetarian diet on telomere length and its correlation with cardiovascular biomarkers in apparently healthy subjects. Therefore, our objectives were to evaluate leukocyte telomere length (LTL) in vegetarians and omnivorous subjects and its association with classical cardiovascular risk biomarkers. From the total of 745 participants initially recruited, 44 omnivorous and 44 vegetarian men apparently healthy were selected for this study and LTL was measured in 39 omnivorous and 41 vegetarians by Real-Time Quantitative PCR reaction. Although telomere length was not different between omnivorous and vegetarians, we found a strong negative correlation between LTL and IMT (intima-media thickness) in omnivorous, but not in vegetarian group. In addition, omnivorous who were classified with short telomere length had higher carotid IMT compared to vegetarians. Our data suggest that telomere length can be a marker of subclinical atherosclerosis in the omnivorous group.

## Introduction

Telomeres are structures of TTAGGG nucleotides repeats at the ends of chromosomes; they prevent the ends of chromosomes from fusing with other chromosomes and from being recognized by proteins of DNA repair as a double-strand break, providing genomic stability and preservation [1].

Because telomere sequences do not fully replicate during DNA replication, they become progressively shorter with each replication of somatic cells [2]. Thus, telomere length is considered a biomarker of biological aging [3] with growing interest in cardiovascular diseases (CVDs) [4–8]. A prospective longitudinal study (average 5.5-yr follow-up) showed that the shortening of telomere length appears to have a predictive value in the incidence and progression of carotid atherosclerosis [4]. Further studies have shown shorter telomere length in subjects with CVDs like atherosclerosis [5], hypertension [6,7], and coronary heart disease [8]. Evidence indicates that, beyond aging, increase of inflammation and oxidative stress accelerates telomere attrition, possibly explaining the association observed between telomere length and CVDs [9,10].

Interestingly, both telomere length and cardiovascular risk can be modified by diet [11–14]. A healthy diet containing high consumption of fruits, vegetables, and whole grains is anti-inflammatory and antioxidative, besides lower severe oxidative stress and DNA damage [15,16]. Lian and collaborators [12] have shown that longer LTL was associated with lower risk of hypertension only in those who consume higher levels of vegetables. In addition, greater adherence to Mediterranean diet was associated with longer telomeres, however, none of the individual dietary components showed an association with telomere length, suggesting that this association may be a consequence of the global effect of this diet.

On the other hand, the high consumption of red/processed meat leads to increased oxidative stress [17–19], which can induce DNA damage and may affect telomere length [20,21]. In this context, a balanced vegetarian diet (which exclude all kinds of meat) would help protect the chromosome ends.

The vegetarian diet is characterized by a high intake of legumes, vegetables, fruits, grains, nuts, seeds, and may or may not include eggs and dairy (milk and milk products) [22]. In general, the vegetarian diet is rich in fibers, vitamin C and E, folic acid, magnesium, phytochemicals, and antioxidants but low in total fat, saturated fatty acid, cholesterol, compared to omnivorous diet [23]. These factors are known to reduce the risk of CVDs [22], and several studies report that vegetarians have a lower cardiovascular risk in comparison with omnivorous subjects [11,24,25]. These findings are in agreement with the typical aspects observed in vegetarian subjects, including lower systolic and diastolic blood pressure, better insulin sensitivity and blood lipids profile [26,27].

Epidemiologic studies have been investigated the telomere length in CVDs, as well as their relationship with diet [8,12,14], but until now, no studies in healthy vegetarian subjects have been done. Given that healthy vegetarian diet is rich in fruits and vegetables, we hypothesized that vegetarian diet can prevent or delay telomere shortening and it can be correlated with lower probability of developing age-related disease, such as CVDs. Therefore, our objectives were to evaluate telomere length in vegetarians and omnivorous subjects and its association with classical cardiovascular risk biomarkers.

## RESULTS

Main characteristics of the study subjects omnivorous (n= 39) and vegetarians (n= 41) are given in Table 1. Higher values of BMI, SBP, DBP, TC, LDL, non-HDL, ApoB, HbA1c, fasting glucose, and IMT were found in omnivorous compared to vegetarians. However, no difference was observed in telomere length between omnivorous and vegetarians (Table 1).

**Table 1:** Clinical parameters of omnivorous and vegetarians subjects.

	**Omnivorous** (n=39) Mean ± SEM	**Vegetarian** (n=41) Mean ± SEM	***P*-value**
**Age (Years)**	47.0 ± 1.50	45.15 ± 1.23	0.341
**BMI (kg/m^2^)**	27.67 ± 0.76	23.31 ± 0.46	< 0.0001***
**SBP (mmHg)**	128.8 ± 2.44	119.8 ± 1.67	0.003**
**DBP (mmHg)**	83.51 ± 1.62	76.32 ± 1.31	0.0009**
**TC (mg/dl)**	200.7 ± 5.58	181.6 ± 6.43	0.029*
**HDL (mg/dl)**	45.28 ± 1.80	47.88 ± 1.47	0.265
**LDL (mg/dl)**	127.1 ± 5.28	111.0 ± 5.29	0.034*
**Non-HDL (mg/dl)**	155.5 ± 5.82	133.9 ± 6.91	0.020*
**TG (mg/dl)**	141.8 ± 10.16	114.2 ± 11.62	0.078
**ApoB (g/l)**	1.007 ± 0.04	0.88 ± 0.04	0.048*
**hsCRP (mg/L)**	3.12 ± 1.06	1.80 ± 0.52	0.065
**HbA1c (%)**	5.56 ± 0.07	5.29 ± 0.04	0.002**
**Fasting Glucose (mg/dl)**	103.3 ± 2.16	94.88 ± 1.17	0.0009***
**IMT (mm)**	668.2 ± 21.12	593.2 ± 14.81	0.004**
**LTL (T/L ratio)**	101.0 ± 5.63	111.2 ± 7.25	0.277

Abbreviations: BMI – body mass index; SBP – systolic blood pressure; DBP – diastolic blood pressure; TC - total cholesterol; HDL – high density lipoproteins; LDL – low density lipoproteins; non-HDL - non-high-density lipoprotein cholesterol; TG – triyglycerides; ApoB – apolipoprotein; CRP – high sensitivity c-reactive protein; HbA1c - Glycated Hemoglobin; IMT - intima-media thickness; LTL – Leukocyte telomere length.

*P<0.05, **P<0.01, ***P<0.001 compared to omnivorous group.

Table 2 shows the correlation of telomere length versus clinical and biochemical parameters. For all the participants, LTL was inversely correlated with SBP, DBP, TC, LDL, and Non-HDL. When we divided the subjects by diet pattern, we found a strong negative correlation between LTL and IMT only in the omnivorous group. In addition, LTL was negatively correlated with TC and non-HDL in vegetarian subjects.

**Table 2:** Correlation between telomere length *versus* clinical and biochemical parameters.

**LTL *vs.***	**All participants**		**Omnivorous**		**Vegetarians**	
	*r*	*P*-value	*r*	*P*-value	*r*	*P*-value
**Age (Years)**	-0.13	0.218	-0.26	0.1	-0.06	0.703
**BMI (kg/m2)**	-0.12	0.275	-0.09	0.57	-0.19	0.232
**SBP (mmHG)**	-0.24	0.028**	-0.28	0.08	-0.17	0.278
**DBP (mmHG)**	-0.25	0.024**	-0.31	0.05	-0.17	0.272
**TC (mg/dl)**	-0.31	0.004**	-0.26	0.10	-0.31	0.045*
**HDL (mg/dl)**	0.17	0.118	0.08	0.61	0.24	0.122
**LDL (mg/dl)**	-0.30	0.005**	-0.23	0.14	-0.26	0.096
**Non-HDL (mg/dl)**	-0.32	0.002**	-0.25	0.12	-0.34	0.027*
**TG (mg/dl)**	-0.19	0.080	-0.09	0.54	-0.20	0.191
**ApoB (g/l)**	-0.24	0.031*	-0.3	0.05	-0.16	0.295
**CRP (mg/L)**	0.007	0.949	-0.15	0.34	0.18	0.260
**HbA1c (%)**	-0.08	0.436	-0.13	0.42	0.05	0.751
**Fasting Glucose (mg/dl)**	-0.15	0.162	-0.09	0.58	-0.04	0.793
**IMT (mm)**	-0.27	0.014*	-0.38	0.01*	-0.02	0.859

Significant at *P<0.05, **P<0.01

Abbreviations: BMI – body mass index; SBP – systolic blood pressure; DBP – diastolic blood pressure; TC - total cholesterol; HDL – high density lipoproteins; LDL – low density lipoproteins; non-HDL - non-high-density lipoprotein cholesterol; TG – triyglycerides; ApoB – apolipoprotein; CRP – c-reactive protein; HbA1c - Glycated Hemoglobin; IMT - intima-media thickness.

Multivariate relationships between LTL and important variables in CVDs are shown in Table 3. For all participants (omnivorous + vegetarians), only the association between LTL and TC remain significantly different. In omnivorous group, the inverse relationship between LTL and IMT was kept. While for vegetarians, there was no longer an association between LTL and the parameters TC, and non-HDL, after including age, BMI, SBP, TC, HbA1c and IMT as independent variables in multivariate analysis.

**Table 3:** Multivariate relationships.

**LTL *vs.***	**All participants**		**Omnivorous**		**Vegetarians**	
	β	*p-*value	β	*p-*value	β	*p-*value
**IMT (mm)**	-0.06	0.172	-0.16	0.018*	-0.01	0.870
**Age (Years)**	-0.10	0.867	0.20	0.786	-0.39	0.703
**BMI (kg/m^2^)**	-0.58	0.681	2.59	0.155	0.31	0.918
**SBP (mmHG)**	-0.27	0.492	-0.13	0.776	-0.13	0.872
**HbA1c (%)**	7.0	0.641	1.48	0.930	19.10	0.534
**TC (mg/dl)**	-0.26	0.046*	-0.13	0.428	-0.35	0.110

Dependent variable: telomere length (TL). Significant at *P<0.05.

Abbreviations: IMT - intima-media thickness; BMI – body mass index; SBP – systolic blood pressure; HbA1c - Glycated Hemoglobin; TC - total cholesterol; β- standardized regression coefficients, obtained from multiple linear regression analysis regression models.

**Figure 1:**
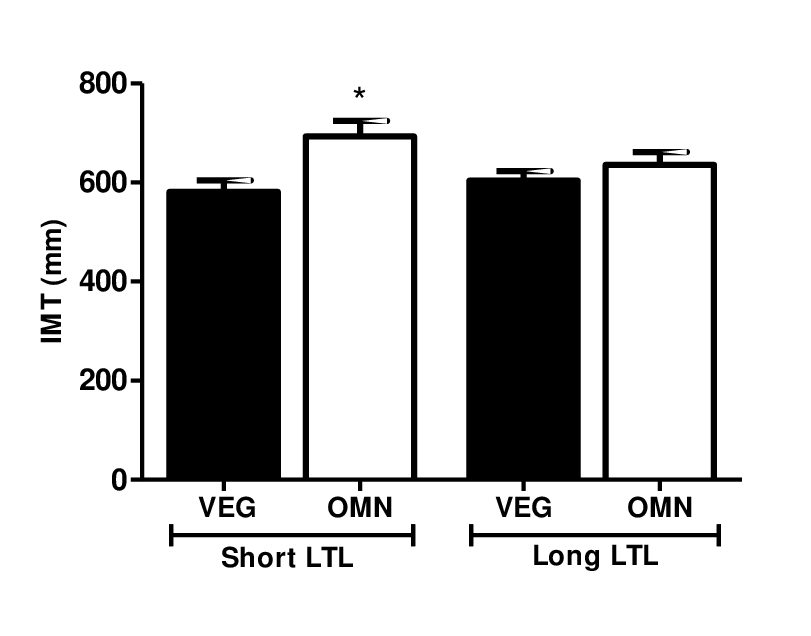
**Carotid IMT level in relation to short and long telomere length.** Data are presented as median and standard error. Omnivorous with short telomere length had higher carotid IMT compared to vegetarians. Significant at *P<0.05 compared to omnivorous with short LTL (*ANOVA, Kruskal-Wallis test, GraphPad Prism software*).

Based on these findings, we compared the carotid IMT measurements in subjects with short and long LTL (Figure 1). Interestingly, omnivorous who were classified with short telomere length had higher carotid IMT compared to vegetarians (581.1 mm ± 23.02 *vs.* 693.3 ± 31.04 mm, *P*= 0.019).

## DISCUSSION

This is the first study that investigated leukocyte telomere length and its association with cardiovascular risk in healthy vegetarian men. There was no significant difference in telomere length between apparently healthy omnivorous and vegetarian men. The main finding of this observational cross-sectional study was the negative association between IMT and telomere length in omnivorous, but not in the vegetarian group. Interestingly, among individuals with short telomere length, a greater IMT rate was observed in omnivorous compared to vegetarians. In vegetarians, lower TC and HbA1c was first correlated with short LTL, however, after adjustments in the multiple regression analysis, this relationship did not remain significant.

Only one study in the literature reported information about LTL in vegetarians [8]. However, this study was addressed to verify LTL in Indian individuals with coronary artery disease (CAD) and the authors only reported that there was a trend towards longer telomeres in vegetarian subjects compared to those who had a mixed diet, but this difference was not statistically significant [8].

Previous studies have shown that shortened LTL can be a powerful marker of increased carotid artery intima medial thickness (IMT) [28,29]. IMT is a non-invasive image method for evaluating modification of functional and structure of the vascular wall, which is important in the development of cardiovascular diseases, such as atherosclerosis [30]. The Framingham Heart Study including 1062 individuals, observed an inverse association of LTL with IMT in obese men, but not in non-obese [28]. In agreement with our finding in omnivorous group, a prospective longitudinal study (over 6 year of follow-up), showed that the shortening of telomere length was associated with increased incidence of subclinical carotid vascular damage (assessed by IMT) in the general population, specifically in men; moreover, subjects with LTL shortened over time had a higher risk of incident cardiovascular events, compared to those in whom LTL lengthened [29]. Another study also suggests that besides being an aging marker and of atherosclerosis, LTL may be a determining factor in arterial aging, perhaps because short telomeres might decrease replicative potential and compromise vascular repair, contributing to the atherosclerotic process [2].

Previously, a cross-section study including healthy individuals over the age of 50 years showed that vascular dilatory functions in vegetarians were better than those omnivorous, and that these effects were dependent of diet and independent from others factors of atherosclerosis, such as smoking, diabetes, hypertension, hyperlipidemia and aging [31]. Also, the authors reported that degree vasodilatation responses were correlated with the years of a vegetarian diet, suggesting that vegetarian diet have a direct effect on vascular endothelial [31]. The mechanism underlying the association between LTL and IMT in omnivorous subjects is still unclear. However, we founded increased BMI, SBP, DBP, TC, LDL, non-HDL, ApoB, HbA1c, fasting glucose, and IMT in omnivorous compared to vegetarians. Consistent with our findings, previous studies have shown that omnivorous present higher BMI, SBP, DBP and worse insulin sensitivity and blood lipids profile, compared to vegetarian subjects [26,32]. Besides that, emerging evidence associates shorter LTL with disease risk factors such as altered lipidic profile [33], development of insulin resistance [34], increased blood pressure [35] and carotid IMT [28,29], suggesting a potential role of telomeres in the development of age-related diseases, including CVDs. Thus, it is possible that metabolic consequences identified in omnivorous compared to vegetarian cause changes in the process related to atherosclerosis and telomere shortening. Another possible explanation for this finding includes increased oxidative stress, resulting from the excessive production of reactive oxygen stress (ROS) and/or the failure of the antioxidant defense mechanisms [36,37]. Due to their high content of guanines, telomeric DNA is sensitive to damage by oxidation, and ROS, including hydroxyl radicals, produce single-strand breaks [37]. In contrast, some dietary factors such as antioxidants, vitamin intake and healthy lifestyle were reported to decrease rates of LTL shortening [10]. These factors can improve inflammation, lower ROS, reduce DNA damage, and increase telomerase activity [15,16,38]. On the other hand, the high consumption of red meat and processed meat lead to increased oxidative stress [17–19], which can induce DNA damage and may have an impact on the telomere length [20,21]. Given that vegetarian subjects have higher plasma levels of antioxidants (ex. carotenoids, ascorbic acid, and beta-carotene) [39,40], a balanced vegetarian diet could be protective against vascular aging.

The limitations of this study include: 1) this cross-sectional study does not prove causality between the effects of different dietary types and LTL and neither of LTL and IMT, however, the sample power calculation and the inclusion criteria strengthen our conclusions 2) relative restricted number of individuals included in each group; 3) we have not evaluated the mechanisms of the vegetarian diet involved in the biomarkers studied.

## CONCLUSION

Despite there is no significant difference in telomere length, the contribution of LTL in relationship carotid IMT was observed in omnivorous but not in vegetarians, even in apparently healthy individuals. Our data suggest that telomere length can be a marker of subclinical atherosclerosis in the omnivorous group. Further studies are warranted to confirm the impact of the vegetarian diet on modifications of telomere length and its protective role of vascular aging.

## MATERIALS AND METHODS

### Subjects and biochemical measurements

Experimental design and samples are shown in Figure S1. In this observational study, we included only apparently healthy individuals, who were vegetarians at least 4 years (lacto-ovo-vegetarians, lacto-vegetarians or vegans) and omnivorous who consumed any type of meat at least five or more servings per week. From 745 recruited individuals, 416 volunteers were women and were excluded because some parameters could be influenced by differences related to phases of the menstrual cycle. After applying inclusion and exclusion criterions, 241 men were excluded and 44 omnivorous and 44 vegetarian apparently healthy men who participated of the final phase of CARVOS Study (Carotid Atherosclerosis, Stiffness Aortic and Risk Factors in Vegetarians and Omnivorous Subjects) were recruited to the study. To telomere assay, DNA samples from eight subjects were not available. Then the analysis was performed to 39 omnivorous and 41 vegetarians. The volunteer men filled out questionnaires regarding past medical history, dietary preferences, family history, physical activity, educational level, and personal data.

We excluded subjects with a history of diabetes, history of dyslipidemia, history of cardiovascular or cerebrovascular diseases, history of hypertension or intake of antihypertensive medication, smoking and age < 35 years. It also excluded vegetarian less than 3 years and omnivorous who eat meat less than four times per week or didn’t fit the inclusion criteria.

The study was approved by the Institutional Review Board at Heart Institute (InCor), Sao Paulo, Brazil (Protocol number: 3751/12/007), following the principles of the Declaration of Helsinki, and all subjects gave written informed consent.

The characteristics recorded for each participant included age, body mass index (BMI), height, systolic blood pressure (SBP), and diastolic blood pressure (DBP).

Blood samples were collected in tube containing ethylenediaminetetraacetic acid (EDTA), after a 10–12 hours fasting, immediately centrifuged and stored at −80 °C until used to measure laboratory analysis, which included total cholesterol (TC); high density lipoproteins (HDL), low density lipoproteins (LDL); non-high-density lipoprotein cholesterol (non-HDL); triglycerides (TG); apolipoprotein (ApoB); C-reactive protein (CRP), Glycated Hemoglobin (HbA1c), and fasting glucose.

### Carotid IMT assessment

As described by Acosta-Navarro [11], in brief, the functional and anatomical properties of the right carotid artery, evaluated as carotid intima-media thickness (IMT), were assessed using an ultrasound device consisting of a vessel wall echo-tracking system (Wall-Track System, PieMedical, Maastricht, The Netherlands). IMT method was performed in the Heart Institute (InCor), a reference hospital from Sao Paulo, being evaluated by a trained professional doing registered calculations. This method provides a reproducible and reliable measurement suitable for routine practice [41] and has been validated and used in other clinical studies [42–44].

All subjects were submitted to B-mode conventional vascular ultrasound of the extracranial carotid artery. The right common carotid artery was obtained 2cm below the carotid bifurcation, and the following measurements were taken: IMT and diameter; beat-to-beat carotid systolic-diastolic variation; and percentage of that systolic-diastolic variation considered the relative distensibility [42].

### Relative telomere length

Peripheral blood samples were collected in tubes containing EDTA, followed by DNA extraction with a high salt method (Lahiri and Schnabel, 1993). DNA was quantified using a NanoDrop Spectrophotometer Thermo Scientific, Nanodrop 200 model, and diluted to 75 ng in 96 well plates. Relative quantification method, described by Cawthon, was used to measure telomere length (Cawthon, 2002). The telomere reaction proceed for one cycle at 95°C for 10 min, followed by 18 cycles at 95°C for 15 s and 54°C for 2 min and primers used were Tel-1 primer (GGT TTT TGA GGG TGA GGG TGA GGG TGA GGG TGA GGG T) and Tel-2 primer (TCC CGA CTA TCC CTA TCC CTA TCC CTA TCC CTA TCC CTA). The 36B4 reaction proceeded for one cycle at 95°C for 10 min, followed by 30 cycles at 95°C for 15 s and 58°C for 1 min 10 s and primers used were 36B4u (CAG CAA GTG GGA AGG TGT AAT CC), 36B4d (CCC ATT CTA TCA TCA ACG GGT ACA A). Each reaction was performed in triplicate for each sample and results were averaged in further calculations. For PCR reactions, PlatinumTaq (Invitrogen) was used and amplicon formation was monitored using SYBR-Green fluorescent dye (Invitrogen). All PCR reactions and fluorescence measures were carried out in an ABI-7500 real-time PCR machine (ABI). For telomere length quantification, the cycle threshold (Ct) for each telomere and control gene 36B4 PCR reaction were calculated using the ABI software algorithm. The telomere/control gene 36B4 (T/C) ratio reflects the relative size of telomere for each sample. Considering the exponential kinetics of the PCR reaction, this ratio may be expressed as the following equation: 2^-ΔCt^, where –ΔCt=-(Ct telomere - Ct control gene 36B4) of sample n. For group comparisons, 2^-ΔCt^ values for each sample were grouped and analyzed together.

### Statistical analysis

Unpaired Student's t-test was used to compare telomere length between vegetarians and omnivorous. Pearson’s (or Spearman´s) correlation coefficients were used to determine the relationship between telomere length and the parameters clinical, and biochemical. Important variables in cardiovascular disease conditions (such as age, BMI, SBP, HbA1c, TC, and IMT) were included as independent variables in the multiple linear regression analysis. Another analysis was performed between the groups, comparing IMT according to telomere length. Subjects telomere length was categorized by long and short telomeres presence, considering median (103.6) as a cut point. Subjects with telomere length measure lower than cut point were included in the short telomere subgroup and those equal or higher than cut point were included in the long telomere subgroup. ANOVA, Kruskal-Wallis test was used to evaluate IMT level in relation to short and long telomere length between the groups. Statistical analyses were performed using GraphPad Prism 5.0 (GraphPad Software, CA, USA) and Stata version 10.0. *P* values <0.05 were considered statistically significant.

## SUPPLEMENTARY MATERIAL

Supplementary Figure 1
