# Supplementary figures and images for: Shortening telomere is associated with subclinical atherosclerosis biomarker in omnivorous but not in vegetarian healthy men
Source: Aging (Albany NY). 2019 Jul 19;11(14):5070–80. doi: 10.18632/aging.102098 (PMC6682516; doi:10.18632/aging.102098)

## SUPPLEMENTARY MATERIAL

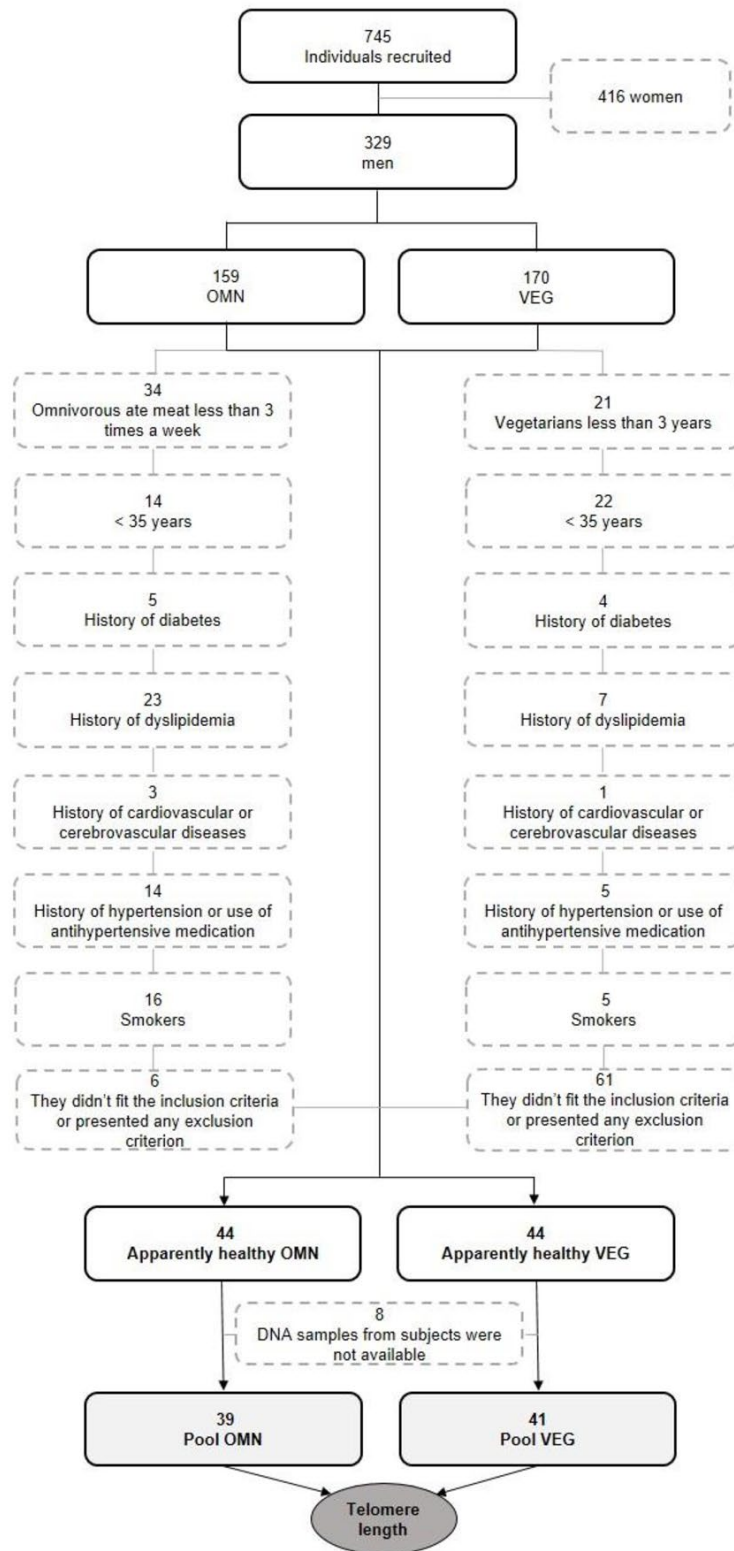

Figure S1. Experimental design and samples.

Supplement: Supplementary Figure 1 [file aging-11-102098-s001.pdf]
